# Regulation of [Ca^2+^]_i_ oscillations and mitochondrial activity by various calcium transporters in mouse oocytes

**DOI:** 10.1186/s12958-020-00643-7

**Published:** 2020-08-15

**Authors:** Feng Wang, Ang Li, Tie-Gang Meng, Le-Yun Wang, Li-Juan Wang, Yi Hou, Heide Schatten, Qing-Yuan Sun, Xiang-Hong Ou

**Affiliations:** 1 Fertility Preservation Lab, Reproductive Medicine Center, Guangdong Second Provincial General Hospital, Guangzhou, 510317 China; 2 China State Key Laboratory of Stem Cell and Reproductive Biology, Institute of Zoology, Chinese Academy of Sciences, Beijing, 100101 China; 3 Department of Veterinary Pathobiology, University of Missouri, Columbia, MO 65211 USA

**Keywords:** Oocyte activation, [Ca^2+^]i oscillations, Mitochondrial membrane potential, Assisted reproductive technology (ART)

## Abstract

Oocyte activation inefficiency is one of the reasons for female infertility and Ca^2+^ functions play a critical role in the regulation of oocyte activation. We used various inhibitors of Ca^2+^ channels located on the membrane, including sarcoplasmic/ endoplasmic reticulum Ca^2+^ATPases (SERCAs, the main Ca^2+^ pumps which decrease the intracellular Ca^2+^ level by refilling Ca^2+^ into the sarcoplasmic reticulum), transient receptor potential (TRP) ion channel subfamily member 7 (TRPM7, a Ca^2+^/Mg^2+^-permeable non-selective cation channel), T-type Ca^2+^ channels and calcium channel Orai1, to investigate their roles in [Ca^2+^]_i_ oscillation patterns and mitochondrial membrane potential during oocyte activation by real-time recording. Our results showed that SERCAs, TRPM7 and T-type Ca^2+^ channels were important for initiation and maintenance of [Ca^2+^]_i_ oscillations, which was required for mitochondrial membrane potential elevation during oocyte activation, as well as oocyte cytoskeleton stability and subsequent embryo development. Increasing the knowledge of calcium transport may provide a theoretical basis for improving oocyte activation in human assisted reproduction clinics.

## Introduction

According to reports by the World Health Organization in 2016, at least one of ten couples in developed countries cannot have children within 5 years of marriage, half of which are due to female infertility [1]. Oocyte activation inefficiency is a major problem causing female infertility [2]. Oocytes are arrested at metaphase of the second meiosis until fertilization takes place. Directly following sperm penetration, oocyte activation begins with a series of crucial steps triggered by periodical repetitive increases and decreases of intracellular Ca^2+^ ([Ca^2+^]_i_) concentrations known as [Ca^2+^]_i_ oscillations [3, 4]. Although [Ca^2+^]_i_ oscillations are required for oocyte activation [5], very little is known about which Ca^2+^ transporter participates in producing [Ca^2+^]_i_ oscillations during oocyte activation.

The importance of Ca^2+^ functions in the regulation of oocyte activation is increasingly being recognized [6]. There are many important channel proteins involved in Ca^2+^ transport in oocytes. Ca^2+^ transporters on the plasma membrane can be controlled by voltage, ligand, second messengers, Ca^2+^ concentration, or other interactions [7]. Ca^2+^ influx into the cytoplasm is mediated by a diverse population of Ca^2+^ transporters exhibiting significant diversities in their gating and activation mechanisms. Once the oocyte is activated, the [Ca^2+^]_i_ oscillations are produced by simultaneous intracellular Ca^2+^ storage release and the extracellular Ca^2+^ influx [8]. Hence, it is important to elucidate the molecular mechanisms of [Ca^2+^]_i_ oscillations at activation. According to our previous research, mitochondria, main organelles for energy production in the cell, are involved in producing [Ca^2+^]_i_ oscillations during oocyte activation [9]. Since Ca^2+^ regulates mitochondria activity in cardiomyocytes [10], we thus believe that [Ca^2+^]_i_ oscillations in the cytoplasm will have similar effects in oocytes. At present, the transporters involved in the regulation of [Ca^2+^]_i_ oscillations and mitochondria activity have not been fully determined.

In this study, specific inhibitors for different Ca^2+^ transporters were introduced to detect the [Ca^2+^]_i_ oscillation patterns and mitochondria membrane potential dynamic changes, in order to evaluate the role of Ca^2+^ transporters in oocyte activation. Studying the function and regulatory mechanisms of these transporters will have significant importance for both understanding oocyte activation mechanisms and for improving clinical assisted reproductive technologies (ART).

## Materials and methods

### Ethics statement

Female ICR mice at 8 weeks old were purchased from the Beijing Vital River Laboratory Animal Technology Co., Ltd. All mice were handled in accordance with the institutional animal care policies of the Institute of Zoology, Chinese Academy of Sciences. Mice were maintained under a 12-h light and 12-h darkness cycle in a specific pathogen-free stage at the Central Animal Laboratory of the Institute of Zoology, Chinese Academy of Sciences. The Laboratory Animal Care and Use Committee of the Institute of Zoology approved this study (The ethics committee approval number: SYXK 2018–0021).

### In vitro transcription of cRNA

The cytoplasmic spindle formation and F-actin meshwork were investigated by live imaging of F-actin with mRNA encoding b5-tubulin–GFP [11, 12] and UtrCH–eGFP [11, 13] as previously described. Templates of in vitro transcription from constructed plasmids were obtained by PCR with F and R primers. PCR products were diluted in RNAse-free water. cRNA transcripts were synthesized in vitro with T7 RNA polymerase mMESSAGE mMACHINE T7 kit (Ambion, Life. Co, Calsbad, CA, USA). Poly(A) tail was added to the sequence end by polymerase tailing kit (PAP5104, Lucigen, Beijing, China). The RNA solutions were then stored at − 80 °C in a final concentration of 400 μg/mL until further use. Approximately 50 pl of RNA solution was injected into each GV oocyte.

### Oocyte collection and partheno-activation

Female mice were injected with 10 IU pregnant mare serum gonadotropin (PMSG, Ningbo Hormone Product Co. Ltd., Cixi, China). After 44 to 48 h, the GV oocytes were collected by ovarian mincing. Female mice were injected with 10 IU PMSG followed 44 to 48 h later by injection of 10 IU human chorionic gonadotropin (hCG, Ningbo Hormone Product Co. Ltd., Cixi, China). After 15 h of hCG, ovulated MII oocytes were collected and denuded in 1 mg/ml hyaluronidase. [Ca^2+^]i oscillations were induced by 10 mM strontium chloride (SrCl_2_, 10,025–70-4, Sangon Biotech, Shanghai, China) in Ca^2+^-free CZB culture medium containing various Ca^2+^ channel modulators for as long as 4 h. After partheno-activation, embryos were moved into KSOM (MR-106, Merck Millipore, USA) for further culture, and pronuclear formation and cleavage were observed.

The details of Ca^2+^ channel blockers used in the study are described below: Thapsigargin (10,522, Cayman, Michigan, USA), a SERCAs inhibitor [14] stored in DMSO in 10 mM, with gradient working concentrations of 0.5, 1, and 10 μM; NS-8593 (N-195, Alomone, Jerusalem BioPark, Jerusalem, Israel), a TRPM7 inhibitor [15], stored in DMSO in 10 mM, with working gradient concentrations of 0.1, 1, and 5 μM; Mibefradil (Mib, HY-15553, MCE, NJ 08852, USA), a T-type Ca^2+^ channels blocker [16], stored in water in 10 mM, with working gradient concentrations of 0.5, 5, and 10 μM; GSK-7975A (HY12507, MCE, NJ 08852, USA), an Orai1blocker [17], stored in DMSO in 10 mM, with gradient concentrations of 10, 100 μM, and 1 mM. All inhibitors were added to Ca^2+^-free CZB incubating medium with an equal concentration of DMSO as the control for 4 h.

### The GV oocyte microinjection

The M2 medium and M16 medium containing 2.5 μM milrinone were prepared and warmed to 37 °C. Milrinone is a phosphodiesterase inhibitor that maintains meiotic arrest once oocytes are removed from the follicles [18]. Micro-drops each containing 20 μl M16 medium with milrinone were prepared in a dish and overlaid with mineral oil. Injection pipettes were made by pulling borosilicate-glass capillary with filament in a mechanical puller. We used a Flaming-Brown micropipette puller (Model P-97) with the following settings: *P* = 540, Heat = 300, Pull = 130, Vel = 100, Time = 150. Microinjection platform was prepared by placing a 10 μl micro-drop of M2 medium with milrinone on a chamber slide, and the drop was covered with mineral oil and placed on the microscope stage. Injection and holding pipettes were placed into the drop of M2 medium with milrinone. cRNA microinjection of GV oocytes was performed with Narishige micromanipulators (Narishige Inc., Sea Cliff, NY) under a Nikon TE 200 (Nikon UK Ltd., Kingston upon Thames, Surrey, UK) and finished within 30 min. After cRNA injection, the oocytes were arrested at the GV stage in M16 medium containing 2.5 μM milrinone for 4 h. Then the oocytes were transferred to M16 medium and cultured under mineral oil at 37 °C, in an atmosphere of 5% CO_2_ in air for 14 h in vitro maturation.

### Real-time recording of [Ca^2+^]i changes

Oocyte [Ca^2+^]_i_ oscillations were detected using 2 μg/mL Fluo-4 AM (488 nm excitation, 525 nm emission) in the partheno-activation solution. Real-time images of [Ca^2+^]_i_ changes after partheno-activation of oocytes which were co-incubated with various Ca^2+^ channel modulators were obtained using a time-lapse confocal laser microscope (UltraVIEW-VoX; PerkinElmer, MA, USA) and recorded at 2 frames per minute. [Ca^2+^]_i_ intensity was detected using an argon laser. Volocity Software was used to analyze fluorescence intensity.

### Real-time recording of oocyte mitochondrial potential

In our study, we set out to confirm the [Ca^2+^]_i_ oscillations regulatory function in mitochondrial activity during oocyte activation. Oocytes were denuded and pre-incubated in M2 culture medium (M7167, Sigma-Aldrich, USA) with 2 μg/mL JC-1 (C2005, Beyotime, Beijing, China), a cell permeable voltage-sensitive fluorescent mitochondrial dye, for 15 min. In order to reduce the toxicity of JC-1 for long-term observation using laser confocal microscope, we reduced the concentration of JC-1 to 0.5 μg/ml in the activating solution. Briefly, the lower potential mitochondria were imaged as JC-1 monomer emitting in green (488 nm excitation, 561 nm emission), and energized high potential mitochondria were imaged in aggregated JC-1 that emits in red (488 nm excitation, 561 nm emission) as fluorescence transporters. Oocyte mitochondrial potential dynamic changes after treatment with various Ca^2+^ channel modulators were recorded after partheno-activation using a time-lapse confocal laser microscope (UltraVIEW-VoX; PerkinElmer, MA, USA) at identical magnification and gain settings throughout the experiments. A software Volocity was used to analyze fluorescence intensity.

### Statistical analysis

All experiments were conducted at least three times. At least 40 oocytes were collected and examined in each experiment and experiments were repeated more than three times. More than a total of 120 oocytes were examined in each group. We presented information of samples with Means and Standard Deviations (SD). Results were analyzed by SPSS 19.0. The significance of differences among groups was analyzed by the Chi-square test, and *p* values less than 0.05 were considered statistically significant.

## Results

### Inhibitors of Ca^2+^ channels affect the efficiency of oocyte activation

We firstly examined the dose-dependent effects of inhibitors of SERCAs, TRPM7, T-type Ca^2+^ channels and Orai1 on the efficiency of SrCl_2_-induced oocyte partheno-activation and found a suitable concentration. The percentages of survival oocytes showed clear cell membrane and high cytoplasmic refraction after activation, pronuclear formation and 2-cell cleaved embryos as shown in Table 1. We applied Thapsigargin (Tha) with gradient concentrations of 0.5, 1, and 10 μM. When its concentration rose to 10 μM, [Ca2+]i would rise slowly, and oocytes died during the activation process. NS-8593 was applied with gradient concentrations of 0.1, 1, and 5 μM. The oocyte survival rate was 37.5% when treating with 1 μM NS-8593. When treating with 5 μM NS-8593, all oocytes died in a short time. Mibefradil was added with gradient concentrations of 0.5, 5, and 10 μM. Under 10 μM, activation of most oocytes was blocked. When applying GSK-7975A, an Orai1blocker, with gradient concentrations of 10, 100 μM, and 1 mM, oocytes were still activated at concentrations of 10 μM and 100 μM GSK-7975A. Pronuclear formation was suppressed significantly when GSK-7975A reached 1 mM, but this concentration had severe cytotoxicity. We found that SERCAs, TRPM7 and T-type Ca^2+^ channels played an important role in activation, while inhibition of Orai1 did not affect oocyte activation. In our subsequent experiments, we selected a moderate concentration of inhibitors to observe the effect of [Ca^2+^]_i_ oscillations on mitochondrial dynamic potential.

**Table 1:** Effect of calcium channel inhibitors on oocyte parthenogenetic activation

Inhibitor	Concentration	No. oocytes	Repeats	PA	PN	2-Cell
Ctrl		240	5	98.33 ± 1.24%	93.25 ± 5.32%	82.90 ± 7.53%
Tha	0.5 μM	240	5	84.2 ± 5.72%	65.7 ± 9.56%*	67.6 ± 7.59%
	1 μM	240	5	66.2 ± 9.24%	42.1 ± 8.56%*	26.1 ± 12.45%*
	10 μM	240	5	0	X	X
NS-8593	0.1 μM	240	5	83.3 ± 6.28%	75.2 ± 9.57%	86.7 ± 7.50%
	1 μM	240	5	37.5 ± 12.53%*	52.9 ± 7.84%*	57.7 ± 11.56%*
	5 μM	240	5	0	X	X
Mib	0.5 μM	240	5	77.3 ± 5.74%	55.6 ± 10.60%	67.5 ± 8.82%
	5 μM	240	5	36.7 ± 5.68%*	42.5 ± 5.93%*	66.4 ± 10.34%
	10 μM	240	5	3.6 ± 2.40%*	0	X
GSK-7975	10 μM	240	5	95.4 ± 3.21%	84.5 ± 6.34%	82.7 ± 7.26%
	100 μM	240	5	83.8 ± 5.21%	82.6 ± 5.83%	58.4 ± 11.57%
	1 mM	240	5	63.5 ± 7.95%*	0	X

Note:Tha indicates SERCAs inhibitor Thapsigargin. NS-8593 is a TRPM7 specific inhibitor. Mib indicates T-type Ca^2+^ channels inhibitor Mibefradil. GSK-7975A is an Orai1 specific inhibitor. PA indicates survival embryos of all activated oocytes after activation. PN indicates pronuclear embryos from surviving oocytes. 2-Cell indicates cleaved embryos in pronuclear fertilized eggs. “X” indicates none available data. The significance of differences between Inhibitors and Ctrl group were analyzed by the Chi-square test and *p* < 0.05 (*) was considered statistically significant

### Dynamic changes in [Ca^2+^]_i_ and mitochondrial potential during oocyte activation

**Fig. 1:**
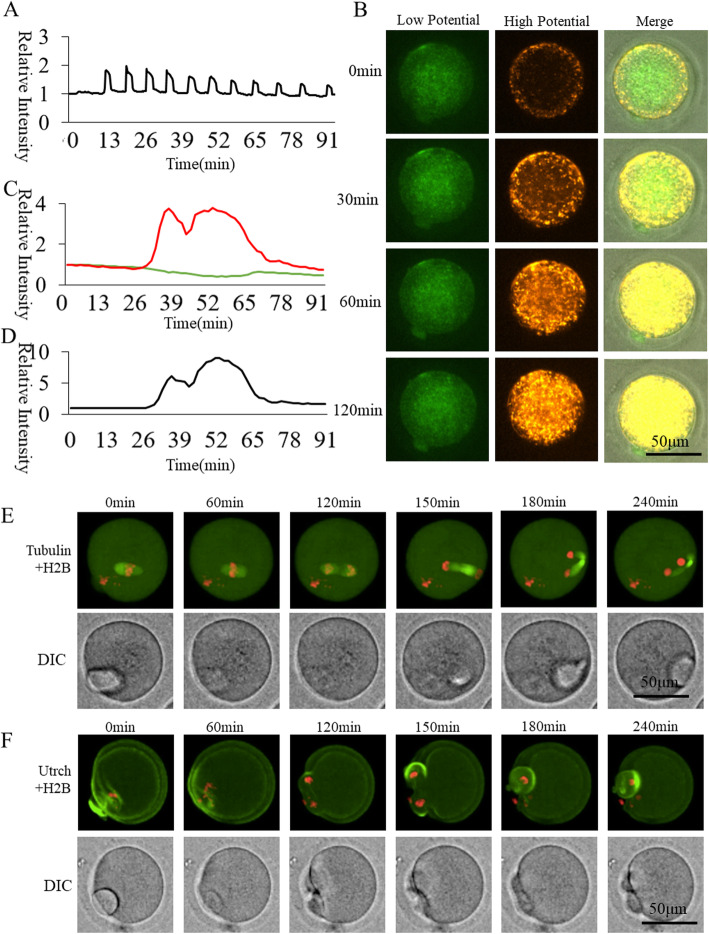
[Ca^2+^]i and mitochondrial membrane potential of wildtype activated oocytes. **a** Cytoplasmic calcium concentrations ([Ca^2+^]_i_) showing dynamic changes in wildtype oocytes during activation. **b** Oocyte mitochondrial membrane potential of wildtype oocytes during activation. **c** Membrane potential fluorescence intensity during oocyte activation. The green and red curves represent labeling with JC-1, indicating relative fluorescence intensities of low membrane potential (488 nm excitation, 525 nm emission) and high membrane potential (561 excitation, 590 emission), respectively. **d** The black curve shows the ratio of high membrane potential to low membrane potential indicating relative mitochondrial membrane potential. **e** Living cell observation of Ctrl oocyte spindle formation **f** Living cell observation of subcortical F-actin distribution in Ctrl oocyte

Cytoplasmic Ca^2+^ concentration ([Ca^2+^]_i_) oscillations and mitochondrial dynamic potential changes during the activation of oocytes were observed by time-lapse confocal laser microscope (UltraVIEW-VoX, PerkinElmer, Massachusetts, USA). Normal [Ca^2+^]_i_ oscillations were induced by SrCl_2_ stimulation (Fig. 1a). We found that mitochondria were activated from the cortex to the internal area of the oocyte and finally aggregated around the chromosomes (Fig. 1b). In order to analyze changes in oocyte mitochondrial membrane potential, a relative fluorescence intensity analysis and a high membrane potential/low membrane potential ratio were introduced. Ordinate was marked as Relative Fluorescence Intensity (relative to the fluorescence intensity of the start point). The overall membrane potential showed two elevated peaks and then returned to a basal level (Fig. 1c, d). Oocyte cytoskeleton (spindle in Fig. 1e, and f-actin in Fig. 1f) showed a clear dynamic distribution during activation.

### Effect of SERCAs inhibitor Thapsigargin on oocyte activation

**Fig. 2:**
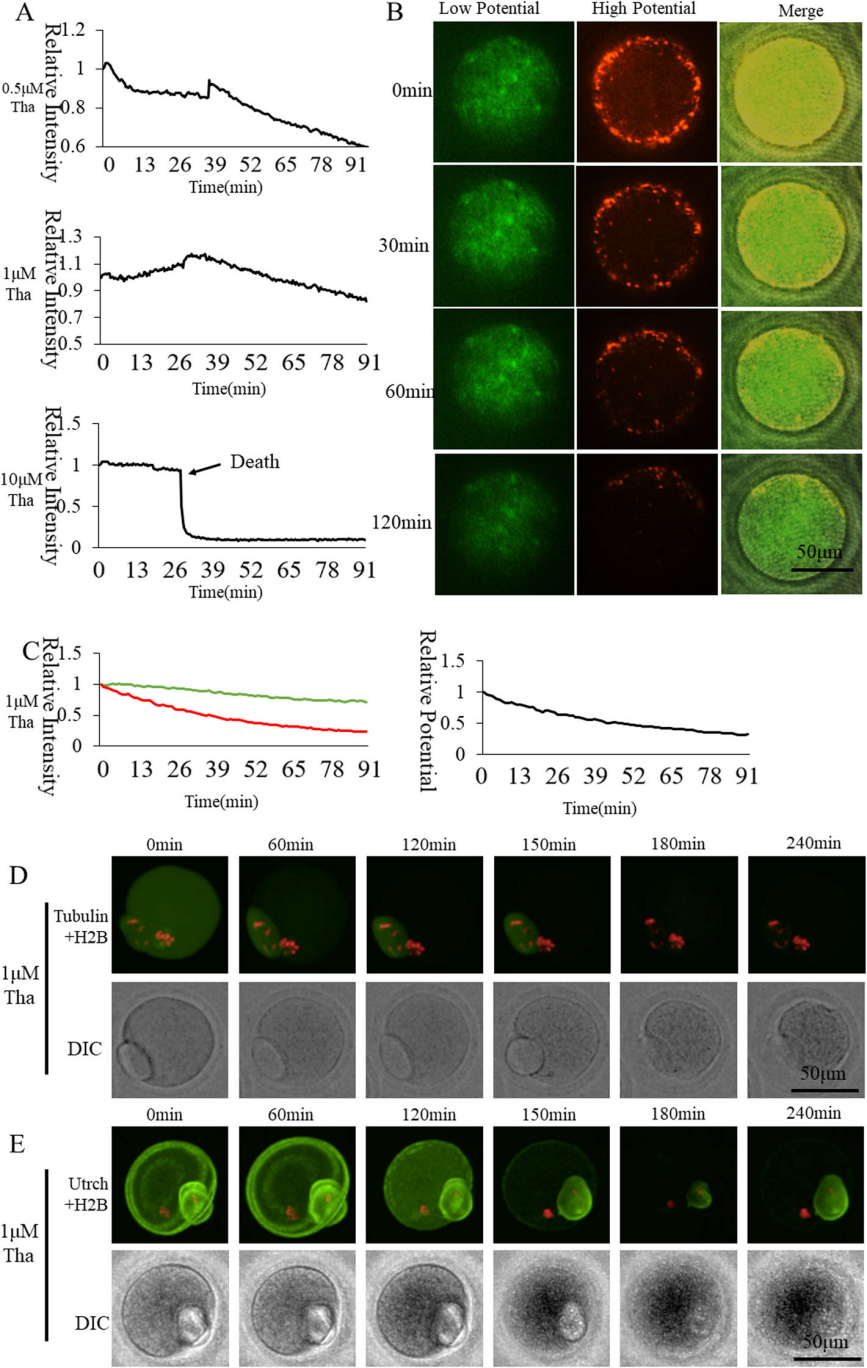
Effect of SERCAs inhibitor Thapsigargin on oocyte activation. **a** Cytoplasmic ([Ca^2+^]_i_) dynamic changes of Thapsigargin-inhibited oocyte during activation. **b** Mitochondrial membrane potential of 1 μM Thapsigargin-inhibited oocytes during activation. **c** Mitochondrial membrane potential fluorescence intensity of 1 μM Thapsigargin-inhibited oocytes. The green and red curves represent labeling with JC-1, indicating relative fluorescence intensities of low membrane potential and high membrane potential, respectively. The black curve shows the ratio of high membrane potential to low membrane potential indicating relative mitochondrial membrane potential. **d** Living cell observation of 1 μM Thapsigargin inhibition of oocyte spindle formation **e** Living cell observation of 1 μM Thapsigargin inhibition of oocyte subcortical F-actin distribution

Endoplasmic reticulum (ER) is a major Ca^2+^ storage component in the cytoplasm. SERCAs are the main transporters for cytoplasmic Ca^2+^ refilling into the ER. We wanted to observe changes in [Ca^2+^]_i_ oscillations and mitochondrial activity during oocyte activation after the Ca^2+^ refill was blocked by SERCAs inhibitor Thapsigargin (Tha) (Fig. 2). The oocyte survival rate was 66.2% when treating with 1 μM Tha (Table. 1), by which concentration [Ca^2+^]_i_ oscillations (Fig. 2a) and pronuclear formation were sufficiently suppressed. Mitochondrial membrane potential continued to decrease under Ca^2+^ refilling inhibition by 1 μM Tha (Fig. 2b). At a concentration of 10 μM Tha completely inhibited Ca^2+^ refill into ER, and oocyte died during activation (Fig. 2a). Ordinate was marked as Relative Fluorescence Intensity (relative to the fluorescence intensity of the start point). Spindle (Fig. 2d) and F-actin (Fig. 2e) showed dispersion when 1 μM Tha caused oocyte death. Thus, Ca^2+^ refilling into ER through SERCAs plays an important role in [Ca^2+^]_i_ oscillations and mitochondrial activation during oocyte activation.

### Effect of TRPM7 inhibitor NS-8593 on oocyte activation

**Fig. 3:**
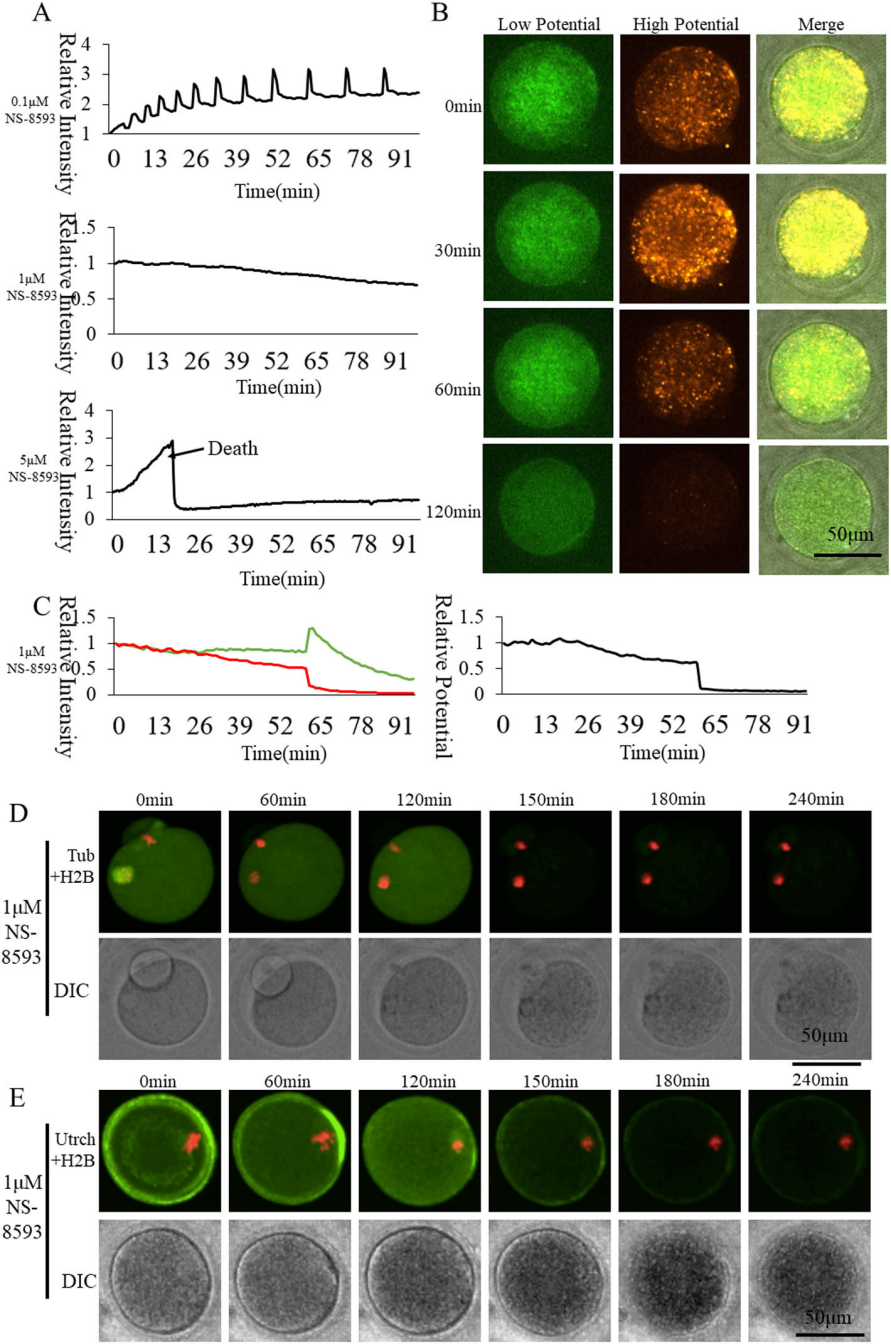
Effect of TRPM7 inhibitor NS-8593 on oocyte activation. **a** [Ca2+]i oscillations of NS-8593-inhibited oocytes. **b** Mitochondrial membrane potential dynamic changes of 1 μM NS-8593-inhibited oocytes. **c** Mitochondrial membrane potential fluorescence intensity of 1 μM NS-8593-inhibited oocytes. The green and red curves represent low and high membrane potential, respectively. The black curve shows the ratio of high membrane potential to low membrane potential indicating relative mitochondrial membrane potential. **d** Living cell observation of 1 μM NS-8593 inhibition of oocyte spindle formation **e** Living cell observation of 1 μM NS-8593 inhibition of oocyte subcortical F-actin distribution

TRPM7 is highly expressed in the GV and MII oocytes, mainly distributed on the plasma membrane. We inhibited TRPM7 activity by its specific inhibitor NS-8593 to study its role in oocyte activation (Fig. 3). The [Ca^2+^]_i_ oscillations (Fig. 3a) and pronuclear formation were sufficiently suppressed when 1 μM NS-8593 was used to inhibit the transport of Ca^2+^ through TRPM7. The dynamic mitochondrial membrane potential of oocytes was observed after treatment with 1 μM NS-8593 (Fig. 3b). At this concentration, mitochondrial membrane potential continuously decreased. Depolarization of mitochondria was found in the case of long-term inhibition (Fig. 3b). When treating with 5 μM NS-8593, [Ca^2+^]_i_ slowly rose and finally induced oocyte death. Ordinate was marked as Relative Fluorescence Intensity (relative to the fluorescence intensity of the start point). Spindle (Fig. 3d) and F-actin (Fig. 3e) showed dispersion when 1 μM NS-8593 caused oocyte death. Thus, [Ca^2+^]_i_ oscillations regulated through TRPM7 are important for mitochondrial activity during oocyte activation.

### Effect of T-type Ca^2+^ channel inhibitor Mibefradil on oocyte activation

**Fig. 4:**
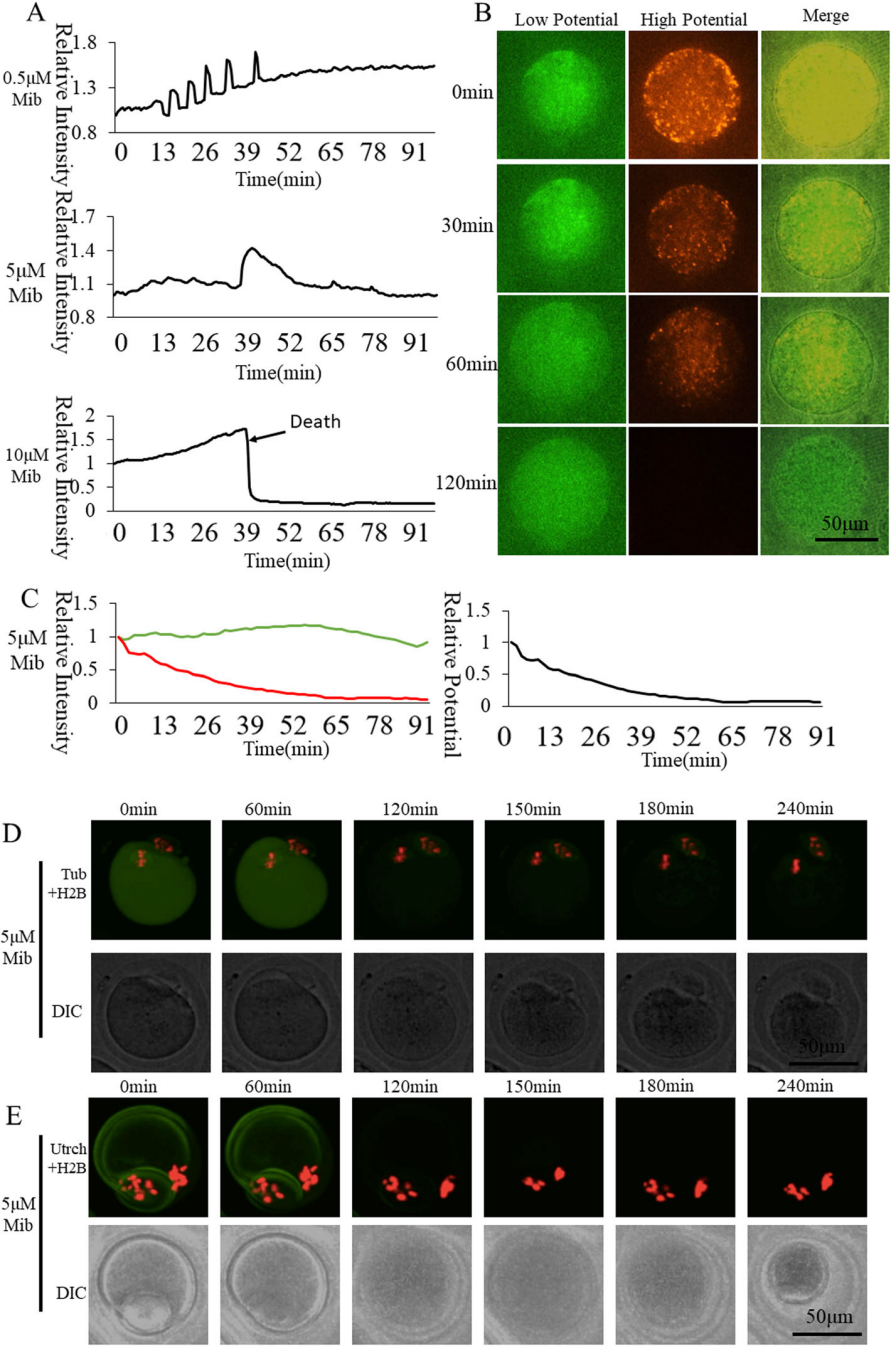
Effect of T-type Ca^2+^ channel inhibitor Mibefradil on oocyte activation. **a** [Ca^2+^]_i_ oscillations of Mibefradil-inhibited oocytes. **b** Mitochondrial membrane potential dynamic changes of 5 μM Mibefradil-inhibited oocytes. **c** Mitochondrial membrane potential fluorescence intensity of 5 μM Mibefradil-inhibited oocytes. The green and red curves represent low and high membrane potential, respectively. The black curve shows the ratio of high membrane potential to low membrane potential indicating relative mitochondrial membrane potential. **d** Living cell observation of 5 μM Mibefradil inhibition of oocyte spindle formation **e** Living cell observation of 5 μM Mibefradil inhibition of oocyte subcortical F-actin distribution

T-type Ca^2+^ channels including Cav3.1, Cav3.2, and Cav3.3 subtypes are distributed on the plasma membrane. Mibefradil can suppress all three T-type Ca^2+^ channels. Mibefradil was introduced to study the role of T-type Ca^2+^ channels in [Ca^2+^]_i_ oscillations (Fig. 4). The mitochondrial membrane potential was observed in the 5 μM group. We found that the mitochondrial membrane potential continued to decrease after inhibiting the transport of Ca^2+^ through T-type Ca^2+^ channels. When the concentration was increased to 10 μM, Ca^2+^ slowly increased, which induced rapid death of most oocytes. Ordinate was marked as Relative Fluorescence Intensity (relative to the fluorescence intensity of the start point). Spindle (Fig. 3d) and F-actin (Fig. 3e) showed dispersion when 5 μM Mibefradil caused oocyte death. Based on these results it is concluded that the transport of Ca^2+^ through T-type Ca^2+^ channels is important for [Ca^2+^]_i_ oscillations and mitochondrial activity in oocyte activation and survival.

### Effect of Orai1 inhibitor GSK-7975A on oocyte activation

**Fig. 5:**
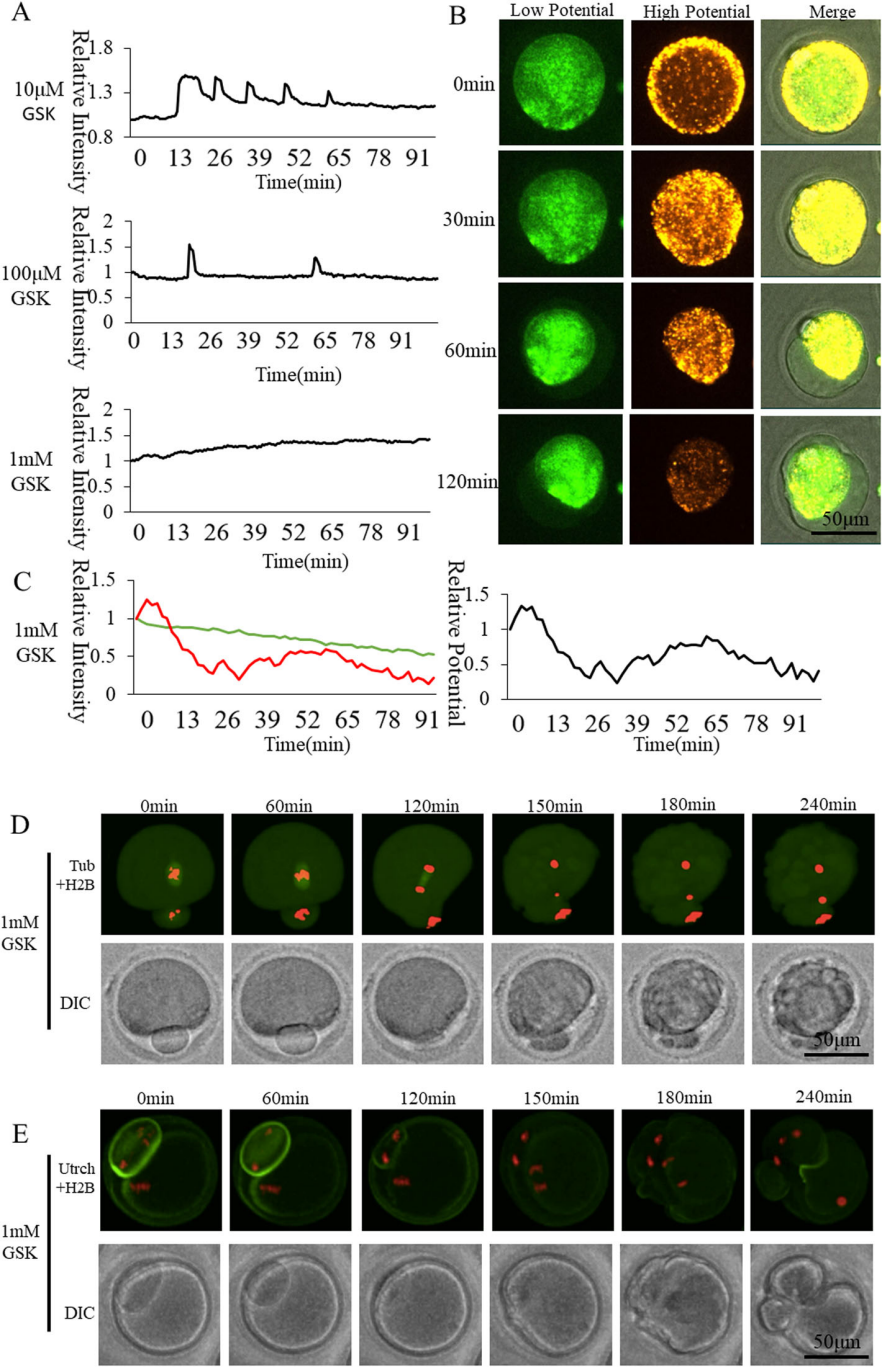
Effect of Orai1 inhibitor GSK-7975A on oocyte activation. **a** [Ca^2+^]_i_ oscillations of GSK-7975A-inhibited oocytes. **b** Mitochondrial membrane potential dynamic changes of 1 mM GSK-7975A-inhibited oocytes. **c** Mitochondrial membrane potential fluorescence intensity of 1 mM GSK-7975A-inhibited oocytes. The green and red curves represent low and high membrane potential, respectively. The black curve shows the ratio of high membrane potential to low membrane potential indicating relative mitochondrial membrane potential. **d** Living cell observation of 1 mM GSK-7975A inhibition of oocyte spindle formation **e** Living cell observation of 1 mM GSK-7975A inhibition of oocyte subcortical F-actin distribution

Orai1 is distributed on the plasma membrane. We studied the role of Orai1 in oocyte activation by treating oocytes with the specific inhibitor GSK-7975A. The mitochondrial membrane potential was observed at 1 mM, which showed irregular changes compared to the Ctrl group (Fig. 5). Most oocytes survived until 4 h of activation, but none of them formed a pronucleus due to cytotoxicity. Ordinate was marked as Relative Fluorescence Intensity (relative to the fluorescence intensity of the start point). It was not confirmed whether this [Ca^2+^]_i_ oscillation pattern and mitochondria membrane potential changes were caused by complete inhibition of Orai1 or by cytotoxicity induced by the high concentration of 1 mM GSK-7975A. Oocytes spindle (Fig. 3d) showed a chromosome segregation and F-actin false distribution when 1 mM GSK-7975A caused oocyte fragmentation. The role of Orai1 in the activation of oocytes requires additional evidence to confirm.

### Effects of Ca^2+^ channel inhibitors on long-lasting [Ca^2+^]_i_ oscillations

**Fig. 6:**
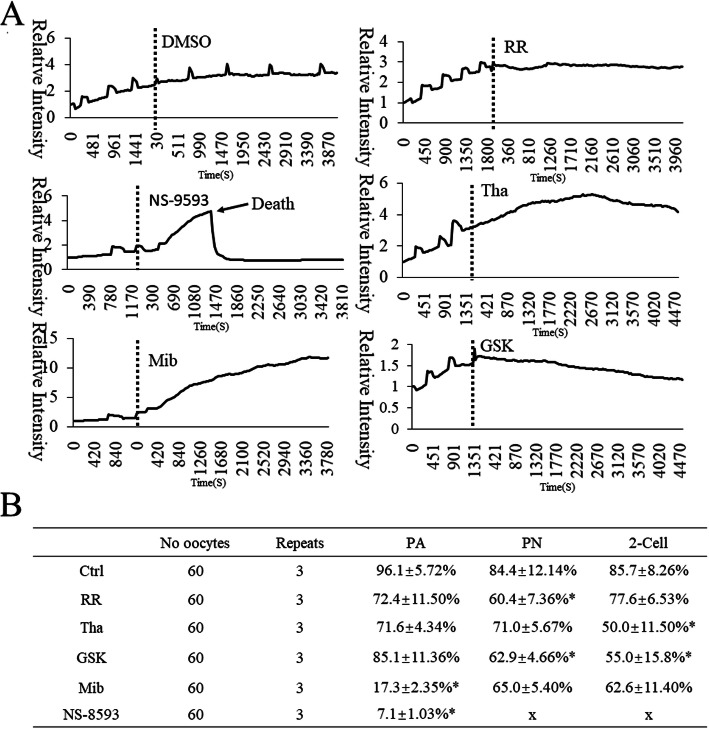
Inhibitor effects on on long-lasting [Ca^2+^]_i_ oscillations. **a** Cytoplasmic [Ca^2+^]_i_ dynamic changes after inhibitor addition. **b** Development of inhibitor addition following [Ca^2+^]_i_ oscillations initiation. PA indicates survival of embryos of all MII oocytes. PN indicates pronuclear embryos of surviving oocytes. 2-Cell indicates cleaved embryos of pronuclear embryos. RR: 10 μM Ruthenium Red; Tha: 1 μM Thapsigargin; GSK: 1 mM GSK-7975A; Mib: Mibefradil; N: 1 μM NS-8593. The significance of differences between groups was analyzed by the Chi-square test and *p* < 0.05 (*) was considered statistically significant. (X) indicates data unavailable

The mechanisms of initiation and maintenance of [Ca^2+^]_i_ oscillations are believed to be different. However, it is unclear which transporters participate in the initiation or maintenance. The effects of several transporters on the maintenance of [Ca^2+^]_i_ oscillations were investigated in our study (Fig. 6). By using time-lapse confocal laser microscope we observed the effect of various inhibitors on the maintenance of [Ca^2+^]_i_ oscillations after some initial oscillations (Fig. 6a). The same concentration was applied as in the mitochondrial membrane potential observation study mentioned above. Ruthenium Red (MACKLIN, Beijing, China) completely inhibited oocyte activation as a non-specific cation channel inhibitor. It was found that the addition of Ruthenium Red, Thapsigargin or GSK-7975A inhibited Ca^2+^ transport, and the [Ca^2+^]_i_ oscillations were blocked from the time of inhibitor addition. After addition of Mibefradil or NS-8593, the intracellular Ca^2+^ increased continuously, and the cell death rate was higher than in the Ctrl and other groups. Based on these results it is concluded that the three Ca^2+^ transporters, SERCAs, TRPM7 and T-type Ca^2+^ channels, are not only involved in [Ca^2+^]_i_ oscillation initiation but also in [Ca^2+^]_i_ oscillations maintenance. Interestingly, the effect of Ruthenium Red and other inhibitors added into culture of oocytes which had already initiated [Ca^2+^]_i_ oscillations in oocytes did not significantly influence oocyte activation (Fig. 6b), suggesting that oocyte activation and pronuclear formation require only a small amount of [Ca^2+^]_i_ oscillations. The function of long-lasting [Ca^2+^]_i_ oscillations in embryo development requires further study.

## Discussion

Obese, diabetic and aging women typically suffer from abnormal body metabolism such as hypertension, hyperglycemia and hyperlipidemia [19–24], causing long-term stress in oocytes [25], which severely damages the quality of oocytes, thereby leading to lower pregnancy rates [26]. Insufficient oocyte activation and mitochondrial damage were considered to be major causes for embryo developmental disorders [27, 28].

Ca^2+^ is one of the major signal molecules that regulate various cell functions including cell cycle progression, arrest and apoptosis. Oocyte activation induces a continuous series of oocyte intracellular Ca^2+^ concentration ([Ca^2+^]_i_) increases and decreases known as [Ca^2+^]_i_ oscillations, which encode oocyte activation events, including release from the MII arrest, pronuclear formation and the transition to embryo development [29]. Release of Ca^2+^ from internal stores and Ca^2+^ influx from the extracellular matrix induce moderate increases in [Ca^2+^]_i_ levels. The increase in [Ca^2+^]_i_ generally lasts about 2 min. As Ca^2+^ refills back into the ER or causes efflux from the cytoplasm to prepare for the next peak of oscillation, the elevated [Ca^2+^]_i_ will return to baseline levels, resulting in an average of a ten to twenty minutes resting interval. [Ca^2+^]_i_ oscillations will last 5–6 h until pronuclear formation takes place [9]. The repeated elevation and recovery of Ca^2+^ signaling is tightly regulated, and the strictly ordered Ca^2+^ signal will coordinate the interaction of various organelles in the oocyte for its activation. Ca^2+^ transporters and regulators could become potential therapy targets for in vitro fertilization failure.

During cell activation, Ca^2+^ shuttles through the cell, and the transporter is likely to be located near IP3R and downstream organelles [30]; the cell membrane, lysosomes, the nucleus, vesicles and mitochondria may be targets of Ca^2+^ release. Endoplasmic reticulum (ER) is a main intracellular Ca^2+^ store, where the Ca^2+^ concentration increases to 300 or even 1000 μM [31]. The pathway for Ca^2+^ efflux from the ER into the cytoplasm has not yet been well identified. SOCE or Ca^2+^ release-activated Ca^2+^ channels (CRAC), were first described in immune cells where they have been shown to be critical for their function. Accordingly, defects in SOCE in humans are associated with severe immune-deficiencies [32]. In oocytes, the predominant [Ca^2+^]_i_ increase pathway appears to be achieved through store-operated Ca^2+^ entry (SOCE) processes. Ca^2+^ enters the cytosol from the endoplasmic reticulum (ER), which in turn opens one of the ER channels, sarcoplasmic reticulum/ER Ca-ATPase (SERCAs), to transport Ca^2+^ back to the ER [33]. Total cellular Ca^2+^ was estimated by the addition of 10 μM Thapsigargin (Tha), a SERCAs inhibitor, which induced complete release of Ca^2+^ from ER [34]. When ER Ca^2+^ stores had been significantly depleted by Tha, sperm no longer triggered [Ca^2+^]_i_ oscillations [35]. In our study, we used three gradient concentrations of 0.5, 1 and 10 μM of Tha. Ten μM Tha kept oocytes at a higher [Ca^2+^]_i_ and all oocytes died before the end of the activation process (Fig. 2). However, when oocyte [Ca^2+^]_i_ oscillations were suppressed by 1 μM Tha, more than half of the oocytes survived more than 4 h. Under such moderate concentration, the effects of SERCAs on mitochondrial activity can be observed for a long term. Oocyte mitochondrial membrane potential continued to decrease under Ca^2+^ refilling inhibition with 1 μM Tha incubation. Not only [Ca^2+^]_i_ oscillations but also mitochondrial activity were suppressed by Tha that induced blocking of ER Ca^2+^ refilling. Finally, oocytes cannot be activated under SERCAs inhibition.

Recently, a member of the TRP channels family, TRPM7, was found to be expressed in mouse GV, MII oocytes and 2-cell embryos [36]. TRPM7 belongs to the subfamily of melastatin and exhibits a ubiquitous tissue distribution. Trpm7 knock-out caused E14.5 embryonic lethality [37]. Using inhibitor NS-8593 suppression of the transporter hours after activation reduced progression to the blastocyst stage [36]. Oocytes treated with 10 μM NS-8593 and fertilized in vitro display impaired Ca^2+^ oscillations [15]. We applied NS-8593 with gradient concentrations of 0.1, 1, and 5 μM (Tab. 1 and Fig. 3a). After treating with 5 μM NS-8593, [Ca^2+^]_i_ slowly rose, which finally induced oocyte death. Treatment with 1 μM NS-8593 kept the survival rate at 37.5% (Fig. 3), while the [Ca^2+^]_i_ oscillations and pronuclear formation were sufficiently suppressed. Under this moderate concentration of 1 μM NS-8593, the effects of TRPM7 on mitochondrial activity can be observed for a long time. Mitochondrial activity did not exhibit the same activation state as the Ctrl in the case of TRPM7 inhibition with NS-8593. Mitochondrial membrane potential continuously decreased when inhibiting the transport of Ca^2+^ through TRPM7 with 1 μM NS-8593 (Fig. 3). The TRPM7 on the cell membrane of oocytes has a significant effect on the [Ca^2+^]_i_ oscillation patterns in oocytes, and [Ca^2+^]_i_ oscillations achieved through TRPM7 is important for mitochondrial activity and oocyte activation.

Mibefradil is a T-type channel inhibitor. Mibefradil was developed as a cardiovascular hypertension and angina remedy [38]. Mibefradil has been repurposed as an anti-cancer drug [39]. However, its underlying mechanisms are still unclear. The mechanism of the anti-cancer therapy is thought to be via the blockage of Ca^2+^ influx through T-type channels [40]. Mibefradil blocks all three subtypes of T-type channels [41], including Cav3.1, Cav3.2, and Cav3.3, with an IC50 (semi-lethal concentration) of 5.8–7.2 μM. In our study, inhibition of Ca^2+^ release by high concentrations of Mibefradil impaired intracellular Ca^2+^ dynamics and thus affected cell viability. We applied Mibefradil at three gradient concentrations of 0.5, 5, and 10 μM (Tab. 1 and Fig. 4). In order to conduct long term observations, a moderate concentration of 5 μM NS-8593 was selected to study the effects of T-type channels on mitochondrial activity. The mitochondrial membrane potential continued to decrease after inhibiting the transport of Ca^2+^ through T-type Ca^2+^ channels with 5 μM Mibefradil. When the concentration reached 10 μM, Ca^2+^ rapidly increased, which induced rapid death of most oocytes. Thus, transport of Ca^2+^ through T-type Ca^2+^ channels is important for mitochondrial activity and oocyte activation.

Orais represents transmembrane proteins that form highly Ca^2+^-selective channels [42]. Orais has three family members, ORAI1, 2 and 3 [43]. Loss-of-function mutation of ORAI1 caused immune deficiency [44] and dysfunction of thrombus formation [45]. SOCE is also mediated through the ORAI channels at the outer membrane. 10 μM GSK-7975A has been reported to induce maximal inhibition of Ca^2+^ influx in Jurkat T-cells [17]. We applied GSK-7975A at gradient concentrations of 10, 100 μM, and 1 mM. We found that oocytes could not be activated when GSK-7975A below 1 mM was used to effectively inhibit Ca^2+^ influx. Mitochondrial dynamic membrane potential showed irregular changes compared to the Ctrl group (Fig. 5). Most of the oocytes survived up to 4 h post activation even at a concentration as high as 1 mM, but none of them formed a pronucleus. Female mice were fertile after knocking out ORAI1 [15]. It is not yet clear whether this [Ca^2+^]_i_ oscillation pattern and membrane potential changes were caused by complete inhibition of Orai1 or by cytotoxicity induced by the high concentration of 1 mM. The role of Orai1 in the activation of oocytes requires additional evidence to confirm.

The mechanisms of initiation and maintenance of [Ca^2+^]_i_ oscillations are different [46]. However, it is unclear which transporters participate in the initiation or maintenance of [Ca^2+^]_i_ oscillations. The effects of several transporters on the maintenance of [Ca^2+^]_i_ oscillations were investigated in our study (Fig. 6). We found that the addition of Ruthenium Red, Thapsigargin and GSK-7975A all inhibited the maintenance of [Ca^2+^]_i_ oscillations. After addition of Mibefradil and NS-8593, the intracellular Ca^2+^ increased continuously, and the cell death rate was higher than in the Ctrl and other groups. All these results suggest that three Ca^2+^ transporters, SERCAs, TRPM7 and T-type Ca^2+^ channels are involved in both the initiation and maintenance of [Ca^2+^]_i_ oscillations. Interestingly, the addition of Ruthenium Red and other inhibitors into culture of oocytes which had initiated [Ca^2+^]_i_ oscillations did not significantly influence oocyte activation (Fig. 6b), suggesting that oocyte activation required only a small amount of [Ca^2+^]_i_ oscillations.

In summary, we applied ER-associated Ca^2+^ transporter SERCAs inhibitor Thapsigargin, TRPM7 inhibitor NS-8593, T-type Ca^2+^ channels inhibitor Mibefradil, and Orai1 inhibitor GSK-7975A to understand the regulation of [Ca^2+^]_i_ oscillations and mitochondrial activity during oocyte activation, and showed that inhibition of SERCAs, TRPM7 and T-type Ca^2+^ channels caused disturbances in Ca^2+^ signaling, mitochondrial activity and subsequent oocyte activation and embryonic development. Once Ca^2+^ transport is abnormal in oocytes, whether it is at the beginning or halfway, will cause disorders of mitochondrial metabolism, activation of subsequent developmental consequences, finally leading to infertility.

## Data Availability

Some or all data used during the study are available from the corresponding author by request.

## Abbreviations

GV: Germinal Vesicle
GVBD: Germinal Vesicle Breakdown
MII: Metaphase II
[Ca^2+^]_i_: Intracellular Ca^2+^ Concentration
[Ca^2+^]_m_: Mitochondrial Matrix Ca^2+^ Concentration
SERCAs: Sarcoplasmic/endoplasmic reticulum Ca^2+^ATPases
TRPM7: Transient receptor potential (TRP) ion channel subfamily member 7
ER: Endoplasmic reticulum
ART: Assisted reproductive technologies
